# IgM-Antibodies against Phosphorylcholine in Mothers and Normal or Low Birth Weight Term Newborn Infants

**DOI:** 10.1371/journal.pone.0106584

**Published:** 2014-09-30

**Authors:** Anna G. Frostegård, Beatrice G. Sjöberg, Johan Frostegård, Mikael Norman

**Affiliations:** 1 Unit of Immunology and Chronic Disease, Institute of Environmental Medicine, Karolinska Institutet, Stockholm, Sweden; 2 Department of Medicine, Karolinska Institutet and Karolinska University Hospital, Huddinge, Sweden; 3 Department of Clinical Science, Intervention and Technology, Karolinska Institutet, Stockholm, Sweden; University of Milan, Italy

## Abstract

**Objective:**

To determine levels of athero-protective IgM antibodies against phosphorylcholine in mothers and term-born normal or low birth weight infants.

**Approach:**

Twenty three mother-infant pairs were studied, of whom 16 infants were within the normal weight range for gestational age (NGA; 3652[504] g) and 7 were small for gestational age (SGA; birth weight: 2715[255] g), the latter <2SD below the Swedish reference data mean for normal fetal growth. All infants were born at term (mean±SD 40.5±1.1 weeks). Serum was available from 6 mothers with SGA and 14 with NGA infants. Participating mothers were aged 34.0±3.9 years (no difference between groups). Fourteen neonates were boys and seven were girls. Levels of anti-PC IgM were determined by ELISA.

**Results:**

Neonatal IgM anti-PC levels were low (undetectable in 8 infants out of which 3 were SGA) with a median of 76[range 0–2.51] U/ml. Maternal IgM anti-PC levels were significantly higher (median 7198[range: 25.32–656.0]) U/ml) and the proportion of mothers in highest quartile (>75th percentile) was larger in mothers of NGA-infants (43%) vs. those of SGA-infants (0%, p = 0.032).

**Conclusions:**

IgM anti-PC levels are low at birth, which suggests that these antibodies do not play a “housekeeping” role in immune function during fetal life/development, but arise predominately on exposure to external antigens after birth. Furthermore, low maternal IgM anti-PC levels may play a role in placental insufficiency, contributing to poor fetal growth and a small-for-date baby. This preliminary observation may have implications for the future risk of atherosclerosis/cardiovascular disease development in pregnant women and their offspring.

## Introduction

Atherosclerosis, the major cause of cardiovascular disease (CVD), is a chronic inflammatory condition characterized by the presence of activated immune competent cells, such as T-cells and monocytes/macrophages, in atherosclerotic plaques [1]. Phospholipids are highly diversified molecular species ubiquitously expressed in cellular membranes, circulating lipoproteins such as low-density lipoproteins (LDL), and certain pathogens. Phosphorylcholine (PC) has been long recognized as the dominant oxidation-related phospholipid neo-epitope, and is an integral part of the pro-inflammatory phospholipid moiety on oxidized LDL (OxLDL). Interestingly, PC is also present in certain microbial pathogens, thus being a dual danger and pathogen-associated molecular pattern (DAMP/PAMP). Several independent studies have consistently linked low IgM anti-PC levels to the development of adult atherosclerosis and CVD [2]. Recently, we reported that low IgM anti-PC levels independently predict death and co-morbidity in acute coronary syndrome [3]. The CVD-protective mechanisms of anti-PC have been shown in mice to include anti-inflammatory [4] and anti-atheroslerotic properties [5], as well as perhaps inhibition of OxLDL uptake by macrophages [6], [7] and inhibition of cell-death [8]. Finally, anti-PC antibodies have long been known to be anti-infectious [9].

Based on animal data, IgM anti-PC antibodies are postulated to be a part of innate “natural” antibody pool. They are generated constitutively, without antigenic stimulation, and are non-redundant for efficient clearance of apoptotic debris/cells. Infants born low birth weight, especially those born small for gestational age (SGA), are at increased life-long risk of developing CVD [10], [11]. Endothelial dysfunction – a key early marker of accelerated atherosclerosis – has been demonstrated in subjects born small, and persists throughout childhood into adult life. Therefore, endothelial dysfunction that begins *in utero* could be a contributing factor to accelerated vascular ageing [12]–[14]. Studies of animals and monozygous twins indicate that early-onset endothelial dysfunction may result from adverse environmental circumstances before birth e.g. malnutrition, especially late in pregnancy. [15] However, the exact cause of l endothelial dysfunction in infants born small at term is unknown.

In this study, we hypothesized that low levels of IgM antibodies against phosphorylcholine during pregnancy could contribute to early-onset endothelial dysfunction, hence play a potential role in mechanisms underlying the increased CVD risk observed in people born small.

## Subjects and Methods

### Ethics Statement

The study was approved by the Ethics Committee at Karolinska Instututet, Stockholm, Sweden and conformed with the principles of the Helsinki Declaration. Written, informed consent was obtained from all mothers.

Participants were 23 mother-infant pairs; 16 mothers delivered a normal birth weight for gestational age infant (NGA; birth weight 3652±[504] g) and 7 delivered a small for gestational age infant (SGA; birth weight: 2715[255] g, i.e., more than 2SD below the Swedish mean for normal fetal growth). The mothers were aged 34.0[3.9] years, with no difference between groups. Fourteen neonates were boys and seven were girls.

All (primi- and multiparous) mothers were healthy, non-smokers, who were not on any special diet and took no medications. Those with manifest (blood pressure; BP>140/90 mm Hg) or borderline hypertension (diastolic BP>85 mm Hg) before pregnancy, insulin-dependent diabetes mellitus, or with signs of glucose intolerance during pregnancy, were excluded. Gestational age (GA) was determined by early routine ultrasound in all pregnancies. Only infants born at term (GA: 40.5 [SD = 1.1, range 37–41] weeks) were included. Multiple pregnancies (twins, triplets) and infants with congenital infection, chromosomal disorders, malformations or neonatal asphyxia were also excluded, as were infants admitted to the neonatal unit for any reason.

Blood samples were drawn simultaneously from both the mother (4 ml) and her newborn infant (2 ml), either at delivery (cord blood from the infant), or in association with the scheduled metabolic screening test three days after birth. Serum was only available for six mothers with SGA-infants and 14 with NGA-newborns. The IgM anti-PC levels were determined using a commercially available anti-PC kit, with low sensitivity range of 0.5 U/ml, according to the manufacturer's instructions (CVDefine©, Athera Biotechnologies, Stockholm, Sweden). Likelihood Ratio Chi-Square was used to compare mothers with SGA or NGA newborns. Statistical analyses were performed with SAS statistical software system (version 9.1).

## Results

IgM anti-PC levels of newborn infants were very low (median 0,76 [min-max range: 0–2,51] U/ml) or undetectable (n = 8, 3 SGA-infants), consequently we refrained from testing for group differences between NGA and SGA infants.

Maternal IgM anti-PC levels were significantly higher with a median of 71.98 [min-max range: 25.32-656.0] U/ml. There was no significant difference between median or mean values of mothers of NGA-infants versus mothers of SGA-infants. Of the NGA-mothers, 6/14 (43%) had IgM anti-PC levels>75^th^ percentile compared with no SGA-mothers (p = 0.032).

**Figure 1:**
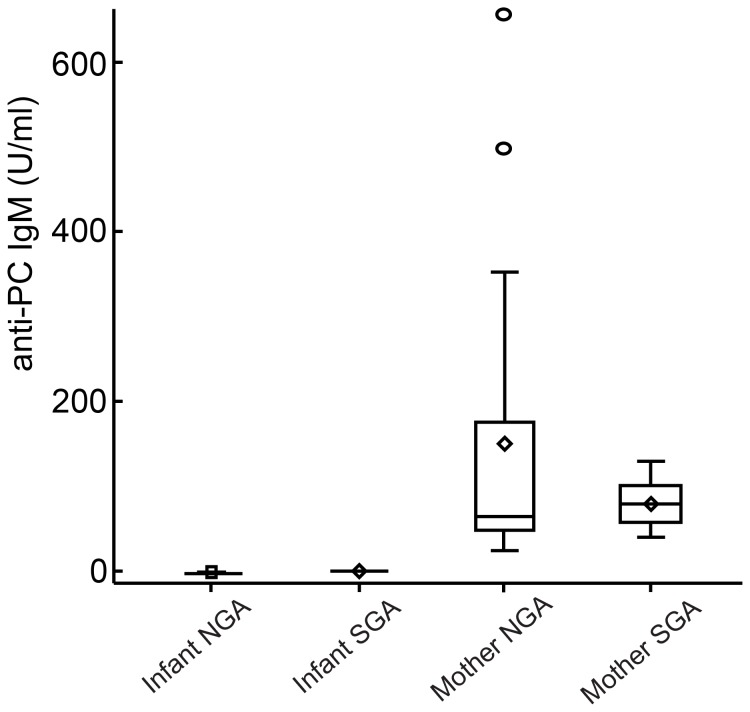
Levels of phosphorylcholine antibodies (IgM anti-PC) among newborn infants with normal (normal for gestational age: NGA, n = 16) or low birth weight (small for gestational age; SGA: birth weight 2 SD or more below the reference mean, n = 7), and in their mothers.

Medians and inter-quartile ranges (IQR) for IgM anti-PC levels in the two groups of mother-infant pairs are presented in table 1, and the distributions of IgM anti-PC levels for the various mother-infant groups are shown in Figure 1.

**Table 1:** Levels of phosphorylcholine antibodies (IgM anti-PC) in newborn infants born at term and in their mothers, stratified by normal (normal for gestational age:NGA, n = 7) or low (small for gestational age:SGA, n = 16)birth weight.

	IgM anti-PC (U/ml)	
*Subjects*	Median	IQR
Infants,NGA	0.48	0.0–0.7
Infants, SGA	0.27	0.0–1.5
Mothers to NGA-infants	62	43–194
Mothers to SGA-infants	79	58–100

IQR = inter quartile range.

## Discussion

This study makes three important observations. Firstly, we found that umbilical or peripheral blood levels of IgM specifically against PC are low-to-undetectable in newborns tested up to 3 days after birth. Maternal levels, by contrast, were comparable to or even higher than previously reported for non-pregnant, slightly older women [3], [4], [7]. Finally, we found that the distributions of IgM anti-PC may differ between mothers of normal birth weight vs. SGA-infants, with a significantly lower proportion of the latter having IgM anti-PC-levels in the highest quartile.

Our findings are in line with recent findings of undetectable anti-PC antibody levels in umbilical blood of newborn infants using an alternative method [16]. The method we used is particularly sensitive, suitable for detecting very low anti-PC levels, which we found in some infants. Our findings are also consistent with a previous study that reported IgM antibodies towards pneumococcal cell wall polysaccharide antigens (which include anti-PC) in only 4–6% of young children [17].

Our finding of strikingly low (nearly “undetectable” levels) levels of IgM anti-PC antibodies in newborns provides further evidence against that the human anti-PC is a “naturally occuring” antibody. Our data do not support the hypothesis that IgM anti-PC antibodies play a role in immune system housekeeping functions very early on [18], at least not during fetal development. Instead, we suggest that human IgM anti-PC may be induced after birth, by exposure to antigens such as PC-exposing microorganisms, rather than being constitutively generated as the immune system develops in utero. This interpretation is supported by our recent study showing that the human anti-PC antibody has undergone somatic mutation, and lacks the clonal properties reported in other species like mice [19].

The hypothesis that anti-PC antibodies play a natural and major role in the process of fast and efficient recognition of apoptotic cell membranes and waste removal-housekeeping is derived principally from studies of mice. [18] A dominant, clonally expanded B-cell subgroup that produces T15 antibodies to PC has been known to exist for some time. T15 has also been shown to play an important role in first-line protection against lethal systemic pneumococcal infection in mice [9]. Furthermore, although mice reared in a germ-free environment produce IgM anti-PC antibodies at some stage during the postnatal period, their IgM anti-PC levels are lower compared with control mice [20]. We are not, however, aware of any data on anti-PC IgM levels of newborn mice.

Our data suggest that murine and human immune responses may indeed differ with respect to PC. Human anti-PC antibodies do not exhibit the murine-dominant T15 anti-PC clonality [19]. One recent study showed that a major proportion of human cord blood IgM binds to OxLDL-related antigens, such as malone-dialdehyde LDL. [20] These antibodies were detected in a semi-quantitative assay, and suggested that these antibodies were natural, generated without previous exposure to external antigens (although no specific PC responses were investigated) [20].

The antibody repertoire of the newborn humans is characterized by a difference between IgG and IgM. Whilst the IgG repertoire of the newborn infant mirrors that of the mother's due to active placental transfer of IgG to the fetus, maternal IgM does not cross the placenta. In contrast, fetal IgM reflects in utero antibody production, independently from the mother. The auto-antibody repertoire is organized in reactivity “networks”, with dominant antigens, a pattern termed immunological homunculus [21]. Natural antibodies against PC are not a part of this repertoire, or of the immunological homunculus.

We have previously reported that anti-PC IgM levels are low in the Swedish population compared with individuals from Kitava (New Guinea), who live a traditional horticultural lifestyle. These data suggest that infections, such as Treponemaor other external antigens, may be necessary to trigger a full anti-PC response [22], [23].

Interestingly, the prevalence of anti-PC levels exceeding the 75^th^ percentile was significantly greater for NGA- compared with SGA mothers. Although this observation must be confirmed in a larger cohort study, it is consistent with our previous report of a negative association between anti-PC IgM levels and atherosclerosis and CVD in adults, and that high anti-PC IgM levels are associated with reduced atherosclerosis [24]. This raises the possibility that maternal anti-PC IgM levels play a role in regulating placental vascular endothelial function during pregnancy, and may perhaps even be involved in the future atherosclerosis/CVD risk of the mother and her child. Whether, and by what pathways, this is linked other underlying factors, such as intrauterine nutrition, is as yet unknown.
